# The Effect of a Multidisciplinary Spine Clinic on Time to Care in Patients with Chronic Back and/or Leg Pain: A Propensity Score-Matched Analysis

**DOI:** 10.3390/jcm11092583

**Published:** 2022-05-05

**Authors:** Ishan Naidu, Jessica Ryvlin, Devin Videlefsky, Jiyue Qin, Wenzhu B. Mowrey, Jong H. Choi, Chloe Citron, James Gary, Joshua A. Benton, Brandon T. Weiss, Michael Longo, Nabil N. Matmati, Rafael De la Garza Ramos, Jonathan Krystal, Murray Echt, Yaroslav Gelfand, Phillip Cezayirli, Neeky Yassari, Benjamin Wang, Erida Castro-Rivas, Mark Headlam, Adaobi Udemba, Lavinia Williams, Andrew I. Gitkind, Reza Yassari, Vijay Yanamadala

**Affiliations:** 1 Spine Research Group, Montefiore Medical Center, Albert Einstein College of Medicine, New York, NY 10467, USA; ishan.naidu@einsteinmed.org (I.N.); jessica.ryvlin@einsteinmed.org (J.R.); devin.videlefsky@einsteinmed.org (D.V.); jonghyun.choi@einsteinmed.org (J.H.C.); chloe.citron@einsteinmed.org (C.C.); james.gary@einsteinmed.org (J.G.); brandon.weiss@einsteinmed.org (B.T.W.); michael.longo@einsteinmed.org (M.L.); rdelag@montefiore.org (R.D.l.G.R.); murrayecht@gmail.com (M.E.); yaroslav.gelfand@gmail.com (Y.G.); philcez84@gmail.com (P.C.); nyassari@wesleyan.edu (N.Y.); benwang2020@gmail.com (B.W.); ryassari@montefiore.org (R.Y.); 2 Department of Epidemiology and Public Health, Albert Einstein College of Medicine, New York, NY 10461, USA; jiyue.qin@einsteinmed.org (J.Q.); wenzhu.mowrey@einsteinmed.org (W.B.M.); 3 Department of Neurological Surgery, Montefiore Medical Center, Albert Einstein College of Medicine, New York, NY 10467, USA; jbenton@montefiore.org (J.A.B.); ecastroriv@montefiore.org (E.C.-R.); mheadlam@montefiore.org (M.H.); audemba@montefiore.org (A.U.); lavwilliam@montefiore.org (L.W.); 4 School of Medicine, Quinnipiac University, North Haven, CT 06518, USA; nabil.matmati@hhchealth.org; 5 Department of Orthopaedic Surgery, Montefiore Medical Center, Albert Einstein College of Medicine, New York, NY 10467, USA; jokrysta@montefiore.org; 6 Department of Rehabilitation Medicine, Montefiore Medical Center, Albert Einstein College of Medicine, New York, NY 10467, USA; agitkind@montefiore.org; 7 Hartford HealthCare, Westport, CT 06880, USA

**Keywords:** multidisciplinary, spine clinic, chronic back pain, leg pain, injections

## Abstract

Chronic back and leg pain are leading causes of disability worldwide. The purpose of this study was to compare the care in a unidisciplinary (USC) versus multidisciplinary (MSC) spine clinic, where patients are evaluated by different specialists during the same office visit. Adult patients presenting with a chief complaint of back and/or leg pain between June 2018 and July 2019 were assessed for eligibility. The main outcome measures included the first treatment recommendations, the time to treatment order, and the time to treatment occurrence. A 1:1 propensity score-matched analysis was performed on 874 patients (437 in each group). For all patients, the most common recommendation was physical therapy (41.4%), followed by injection (14.6%), and surgery (9.7%). Patients seen in the MSC were more likely to be recommended injection (*p* < 0.001) and less likely to be recommended surgery as first treatment (*p* = 0.001). They also had significantly shorter times to the injection order (log-rank test, *p* = 0.004) and the injection occurrence (log-rank test, *p* < 0.001). In this study, more efficient care for patients with back and/or leg pain was delivered in the MSC setting, which was evidenced by the shorter times to the injection order and occurrence. The impact of the MSC approach on patient satisfaction and health-related quality-of-life outcome measures warrants further investigation.

## 1. Introduction

Back and leg pain are leading causes of disability worldwide. Low back pain is the most common musculoskeletal disorder [1,2,3,4] and is also a leading cause of work absenteeism [5]. The economic burden is also substantial, with an estimated direct and indirect cost of USD 100 billion per year in the United States [6]. The initial treatment of back and leg pain without the associated red flags is varied and includes anti-inflammatory medications, muscle relaxants, referrals to physical therapy or pain interventions (such as epidural steroid injections, facet blocks, or nerve blocks), and others [7].

Multidisciplinary approaches have allowed clinicians to treat complex conditions, including spinal disorders, more efficiently [8]. For patients with spinal tumors, multidisciplinary discussions allow treatment recommendations to be tailored to each patient on the basis of their personal and tumor-specific factors in an efficient and systematically optimized fashion [9]. Likewise, multidisciplinary approaches have been found to significantly reduce complications and surgical durations for patients undergoing complex spine surgery [10].

Nonetheless, data on multidisciplinary teams that evaluate patients with back and/or leg pain during the same office visit are limited. At our institution, patients referred to spine surgeons for back and/or leg pain are seen either by the surgeon alone (unidisciplinary spine clinic (USC)), or in a separate multidisciplinary spine clinic (MSC) that includes a co-located cross-disciplinary team of neurosurgeons, orthopedic surgeons, pain-management physicians, and physiatrists. Patients can be evaluated by any of the team members during the same visit. In this manuscript, we compare the differences in the treatment recommendations, the time to the treatment order, and the time to the treatment occurrence for patients seen in a USC versus a MSC setting.

## 2. Materials and Methods

### 2.1. Study Design and Patient Selection

This is a retrospective cohort study. After institutional-review-board approval (IRB 2019-10657), eligible patients were identified through local healthcare surveillance software (Clinical Looking Glass [CLG]; Streamline Health) and an electronic-medical-record (EMR) review. We included adult patients (age 18+) initially presenting to orthopedic spine or neurological spine surgeons for back and/or leg pain at our institution between 1 July 2018 and 30 June 2019. Patients were excluded if they previously sought care for their chief complaint from another spine specialist at our institution, including Emergency Room visits, or if their chief complaint was cervical spine pathology or myelopathy.

### 2.2. Clinic Settings

Our institution’s central call center is responsible for scheduling all spine surgery visits, and for allocating patients to the USC or MSC, which are both located in close proximity in the Bronx, New York. Appointments are assigned on the basis of next availability alone, but the USC has a larger capacity and, thus, a higher number of patients were seen there initially. At the USC, patients are evaluated only by the spine surgeon, and they can receive a specific treatment recommendation, a treatment order, or they can be referred to another specialist within our system. In contrast, the MSC includes four specialties on site: neurosurgery, orthopedic surgery, pain medicine, and physiatry. At the MSC, spine surgeons discuss the case with other team members on site, and patients can be seen by multiple specialists during the same clinic visit, if deemed appropriate.

### 2.3. Assessments and Outcomes

The index visit (baseline) was defined as the initial visit with the spine surgeon during the study period at either the USC or MSC. We collected demographic data on age, gender, race/ethnicity, medical insurance, the Charlson Comorbidity Index (CCI) score, and the diagnosis at baseline. Data were identified and collected in the form of ICD-9 codes.

We assessed the clinical outcomes up to 180 days after the index visit, as this was considered sufficient time for patients to receive a treatment recommendation. Outcomes included:

1. Differences in the first treatment recommendations; this had six possible categories (three actual “treatments”): injection, physical therapy, surgery, medically cleared, lost to follow-up, or no recommendation. Each category was analyzed separately. The “injection” category included epidural steroid injection (ESI), radiofrequency ablation, and/or nerve block. Patients were labelled as “no recommendation” if no treatment recommendation was made within 180 days. Patients were considered lost to follow-up if they did not return to either the MSC or the USC within 180 days of their last visit. If patients received multiple treatment recommendations throughout one year from the index visit, only the initial treatment was used in our analysis. All references to “treatment” in this manuscript hereafter refer to the first treatment that was recommended by the surgeon;
2. Time to treatment order; defined as the time from the index visit to the date when the treatment was ordered in the EMR. The date of treatment was recorded for patients recommended injection and surgery. We could not analyze PT treatment because many patients received PT outside our institution/network. Some treatment orders may not have occurred at day zero if further imaging or insurance authorization was required, for example;
3. Time to treatment occurrence; defined as the time when the ordered treatment was delivered.

### 2.4. Statistical Analysis

A 1:1 propensity score-matched analysis was performed. The propensity score was nonparsimonious and was estimated from a logistic regression model with all the baseline covariates (age, sex, race/ethnicity, insurance, and CCI score). Matching was performed without replacement and with a caliper width of 0.2. Patient characteristics were compared between the USC and the MSC by using Wilcoxon rank sum tests for continuous variables, and Pearson’s chi-squared tests or Fisher’s exact tests for categorical variables. Pearson’s chi-squared tests or Fisher’s exact tests were used to compare the rates of treatment recommendations between the USC and the MSC. Kaplan–Meier survival curves were plotted for time analyses, and log-rank tests were used to compare the curves. Treatment (injection and surgery, separately) rates and lead times were analyzed similarly. Statistical significance was specified a priori as *p* < 0.05. Analyses were conducted in SAS 9.4 and R 3.5.1.

## 3. Results

### 3.1. Patient Characteristics

A total of 1738 patients with a chief complaint of back and/or leg pain were seen by a spine surgeon between 1 July 2018 and 30 June 2019. There were 1004 patients (57.8%) seen at the USC, and 734 at the MSC (42.2%). The diagnoses included low back pain (73.5%), radiculopathy (10.3%), spinal stenosis/neurogenic claudication (4.5%), and others (11.7%).

The baseline patient and socioeconomic characteristics are summarized and compared in Table 1. The average age was 53.3 years for patients seen at the USC, versus 54.0 years for patients seen at the MSC (*p* = 0.332); over two-thirds of the patients in both groups were female (66.1% in the USC vs. 63.2% in the MSC, *p* = 0.227). There were significant differences in the race distribution between groups, with a lower proportion of White and Black patients seen at the USC compared to the MSC (*p* = 0.002). On the other hand, a higher proportion of patients in the USC had Medicaid or were uninsured compared to the MSC (*p* = 0.001). The average CCI score was significantly lower in the USC clinic (1 vs. 0.9, *p* = 0.013).

These differences warranted a propensity score-matched analysis, in which 437 patients in the USC were matched to 437 patients in the MSC; the results are summarized in Table 2. The average ages in the USC and MSC clinics were 54.7 and 54.8 years, respectively (*p* = 0.880). The proportion of females in the USC was 64.1%, and it was 58.4% in the MSC (*p* = 0.096). The racial distribution (*p* = 0.432), the insurance distribution (*p* = 0.162), and the average CCI scores (*p* = 0.671) were no longer significantly different between the two groups.

### 3.2. Treatment Recommendations and Time to Care

For all patients, the most common recommendation was physical therapy (41.4%), followed by injection (14.6%), and surgery (9.7%). The differences in the treatment recommendations and the timing are summarized in Table 3. A significantly higher proportion of patients seen at the MSC were recommended injection (19.2% vs. 10.1%, *p* < 0.001), and a significantly lower proportion of patients were recommended surgery as their first treatment (6.4% vs. 13.0%, *p* = 0.001).

**Figure 1:**
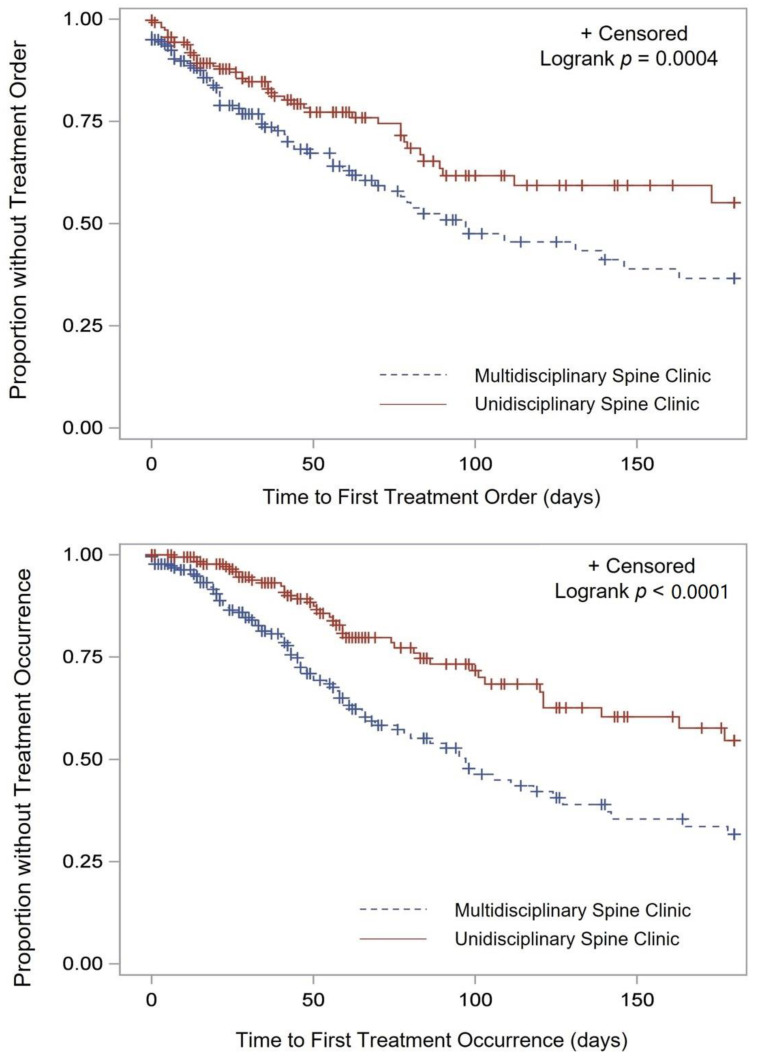
Time to injection order (**top**) and occurrence (**bottom**).

The time to order for injections was significantly shorter for patients seen at the MSC (median of 19 vs. 28 days, *p* < 0.001) (Figure 1).

**Figure 2:**
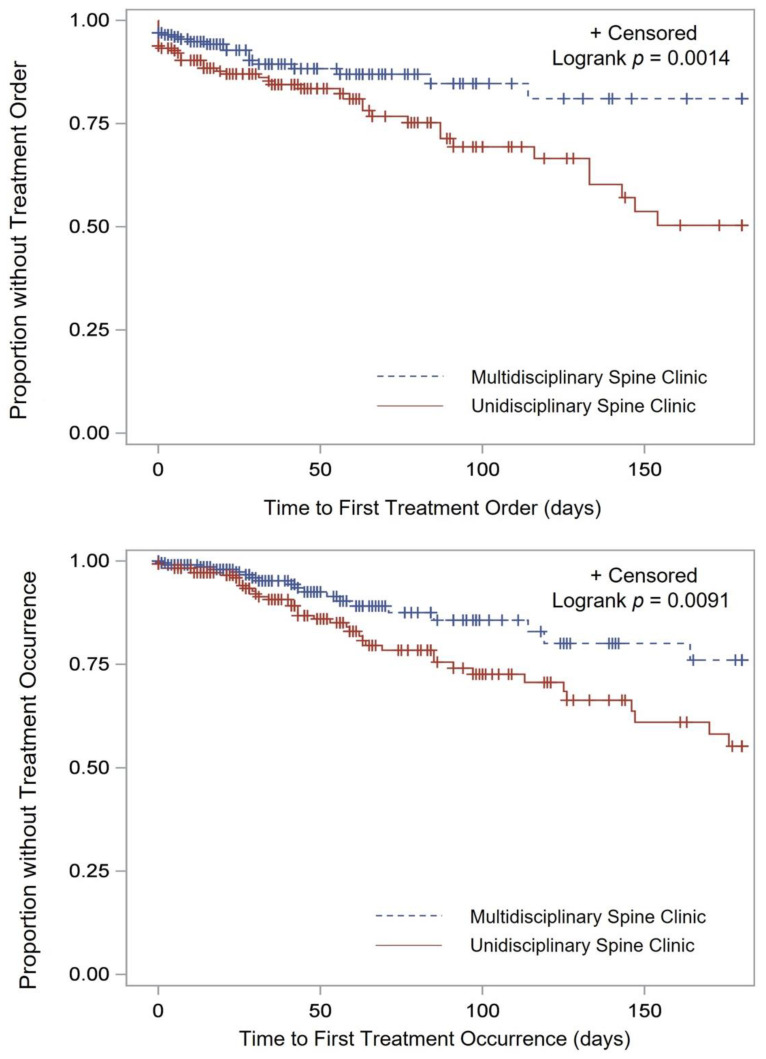
Time to surgery order (**top**) and occurrence (**bottom**).

Additionally, the time it took for patients to actually receive the injection was also significantly shorter (43 vs. 54 days, *p* < 0.001) (Figure 1). The time to the surgery order and the time to receive surgical intervention were longer in the MSC (Figure 2).

## 4. Discussion

Longer times to care delivery have been associated with suboptimal outcomes and decreased patient satisfaction for patients with chronic pain [7,11,12]. In this study, we examined the differences in the treatment recommendations and the time to care for patients with chronic back and/or leg pain who were seen at a USC versus an MSC, and we found that the multidisciplinary setting was more efficient, as was evidenced by the shorter times to the injection order and occurrence. Patients were also less likely to obtain the recommended surgery when evaluated in the MSC; the time to the order and occurrence of surgery were longer for the patients who were seen at the MSC.

### 4.1. Key Findings

The most common initial treatment recommendation for patients seen at both clinic settings was physical therapy. The most common recommended intervention was injections in 14.6% of cases, followed by surgery in 9.7% of cases. Patients seen at the MSC were more likely to be recommended an injection and were less likely to be recommended surgery as a first treatment, compared to patients seen at the USC. Although the exact reason is unknown, the fact that the pain medicine clinician and physiatrist can both see the patient at the same visit may account for this finding. These specialists can offer patients nonsurgical treatment options and can further discuss cases with the surgeon in real time. The most noteworthy finding, however, was the significantly shorter times to the treatment order and the actual treatment occurrence that were seen after patients were evaluated in the MSC, and specifically for injections.

### 4.2. Interpretation and Generalizability

Epidural injections, radiofrequency ablation, and nerve blocks are part of the armamentarium that is used by physicians to treat chronic back and/or leg pain. A survey of 314 practitioners who treated patients with back and radicular pain showed that over 77% recommended lumbar interlaminar injections, and over 97% recommended transforaminal injections [13]. The evidence suggests an improvement in symptoms for up to 3 months after treatment [14]. Furthermore, a phase 3 multicenter randomized controlled trial (NERVES) that compared epidural injection versus surgery (microdiscectomy) for sciatic pain was recently published; no significant differences in the clinical outcomes between treatments were found; however, the complications and the cost of the microdiscectomy were significant [15]. The authors of that study recommended a stepwise approach towards the treatment of leg pain, suggesting that injections would have lower complication rates, be less costly, and achieve similar levels of improved outcomes compared to surgery [15]. For patients with chronic low back pain, fusion surgery has not shown to be superior to conservative management (including injections), but it has been associated with a higher complication rate [16,17].

Timely access to treatments, such as injections, is of the upmost importance for patients who suffer from debilitating back and/or leg pain. In the present study, a significant benefit in the time to the injection order and the injection occurrence was seen for patients evaluated in the MSC setting. Surgery is rarely recommended on the first visit to a spine clinic, and our study suggests that multidisciplinary approaches may decrease surgical recommendations further during the index visit. Having a multidisciplinary team available to discuss a case may allow for the exploration and recommendation of other treatment options.

Commensurate with our findings, other studies have shown significant benefits in the time to care when multidisciplinary approaches are employed in patients with spinal disorders [18,19,20]. Wilgenbusch et al. evaluated the impact of a multidisciplinary spine approach (the “Saskatchewan Spine Pathway” (SSP)) in the evaluation of patients with low back and/or leg pain [19]. This pathway consisted of a referral system in which primary care physicians, chiropractors, physiotherapists, and pain physicians evaluate and treat patients before referring them to a surgeon for consultation. The study found that the patients who were referred to the surgeon via the SSP (as opposed to a direct referral from primary care to the surgeon) were more likely to be candidates for surgery and had significantly shorter wait times for imaging and surgical assessment [19]. This is similar to our findings, in that more efficient care was delivered when a multidisciplinary approach was employed. Although patients referred through the SSP had a higher rate of recommendation for surgery (as opposed to our findings, in which surgical recommendations were lower in the MSC setting), there are several important differences that explain these findings. First, the patients who were referred through the SSP had already been evaluated and had even tried conservative therapy prescribed by appropriate physicians before being seen by the surgeon, which explains why they were more likely to be surgical candidates. Second, the SSP is a referral system rather than an MSC, in which patients are evaluated by multiple specialists at the same time and during the same visit, which is something that is unique to our healthcare system.

A recent single-center prospective cohort study also evaluated the effect of the SSP on patients with low back pain [20]. The authors found shorter wait times for magnetic resonance imaging, a higher utilization of nonoperative treatment strategies before seeing a surgeon, and higher patient satisfaction prior to surgical assessment for patients in the SSP group [20].

Drymalski and Agha described their experience creating the MU (University of Missouri) Comprehensive Spine Center [21]. In their model, patients with back and/or leg pain are also referred to a single call center (similar to our institution) but are initially seen by a physiatrist, who is in charge of directing the nonoperative care and education. If the physiatrist deems that a patient needs to be seen by a surgeon, a rotation is designed to distribute referrals equally amongst neurosurgeons and orthopedic surgeons. Drymalski and Agha describe how this multidisciplinary approach allowed them to improve access to surgical patients and lowered waiting times for evaluation [21]. Although the MU system is different from ours, it highlights the importance of involving other subspecialties when treating patients with back and/or leg pain, and it evidences the more efficient care that can be delivered.

Namiranian et al. also describe their experience with a multidisciplinary spine board for patients with low back pain [22]. The authors established a group to discuss patients “at highest risk of poor outcomes following an elective lumbar spinal surgery” [22]. The team consisted of orthopedic and neurologic spine surgery, pain psychology, physical therapy, radiology, pain pharmacy, primary care, pain management, anesthesiology, and veteran advocacy. They found that, after the implementation of multidisciplinary case discussions, the utilization for elective lumbar spine surgery decreased significantly, which is similar to our findings on the lower surgical-utilization rates in the MSC.

### 4.3. Limitations

The retrospective nature of this study and the lack of other objective health-related quality-of-life outcome measures are important limitations. Although age, sex, race, insurance, and CCI were used for the matching analysis, unmeasured covariates may also impact treatment recommendations and the time to care. A single-center institution is also subject to selection bias, and the population that it treats may not necessarily represent other populations with back and/or leg pain. Nevertheless, the first step was to evaluate whether or not the care was more efficient in the MSC setting; this was evidenced by significantly shorter times to the injection recommendation and occurrence. Given that only the initial visit was examined, whether treatment recommendations or times differed after a second or third visit is also unknown. This also means that the success rate for each of the initial treatments is unknown. Future prospective studies are currently planned to address these limitations.

## 5. Conclusions

Multidisciplinary approaches allow for more efficient healthcare delivery for patients with complex health conditions. Nonoperative care still remains the first-line treatment for patients with chronic back and/or leg pain without any red flags. In this study, patients evaluated in an MSC received more efficient care, which was evidenced by the shorter times to the injection order and the injection occurrence. Our approach is unique in that patients are seen by multiple specialists at the same time, and it does not require referrals or multiple visits. The impact of the MSC approach on patient satisfaction and quality of life warrants further investigation.

## Figures and Tables

**Table 1:** Baseline and socioeconomic characteristics of all patients presenting with back and/or leg pain to the Unidisciplinary Spine Clinic (USC) and Multidisciplinary Spine Clinic (MSC).

Parameter	USC (*n* = 1004)	MSC (*n* = 734)	*p*-Value
Age (mean years, SD)	53.3 (15.7)	54.0 (16.6)	0.332
Male (%)	340 (33.9)	270 (36.8)	0.227
Female (%)	664 (66.1)	464 (63.2)	
Race/ethnicity			
White (%)	58 (5.8)	71 (9.7)	0.002
Black (%)	213 (21.2)	181 (24.7)	
Hispanic (%)	401 (39.9)	260 (35.4)	
Other (%)	55 (5.5)	50 (6.8)	
Unknown/Not reported (%)	277 (27.6)	172 (23.4)	
Insurance			
Medicare (%)	259 (25.8)	213 (29.0)	0.001
Medicaid (%)	361 (36.0)	219 (29.8)	
Private (%)	280 (27.9)	249 (33.9)	
Uninsured (%)	104 (10.4)	53 (7.2)	
CCI (mean score, SD)	0.9 (1.7)	1 (1.7)	0.013

SD: standard deviation, CCI: Charlson Comorbidity Index.

**Table 2:** Propensity score-matched analysis of baseline and socioeconomic characteristics in patients seen at the Unidisciplinary Spine Clinic (USC) and Multidisciplinary Spine Clinic (MSC).

Parameter	USC (*n* = 437)	MSC (*n* = 437)	*p*-Value
Age (mean years, SD)	54.7 (15.2)	54.8 (16.7)	0.880
Male (%)	157 (35.9)	182 (41.6)	0.096
Female (%)	280 (64.1)	255 (58.4)	
Race/ethnicity			
White (%)	35 (8.0)	48 (11.0)	0.432
Black (%)	100 (22.9)	112 (25.6)	
Hispanic (%)	166 (38.0)	151 (34.6)	
Other (%)	32 (7.3)	30 (6.9)	
Unknown/Not reported (%)	104 (23.8)	96 (22.0)	
Insurance			
Medicare (%)	130 (29.7)	126 (28.8)	0.162
Medicaid (%)	145 (33.2)	122 (27.9)	
Private (%)	124 (28.4)	153 (35.0)	
Uninsured (%)	38 (8.7)	36 (8.2)	
CCI (mean score, SD)	1.1 (1.8)	1.1 (1.7)	0.671

SD: standard deviation, CCI: Charlson Comorbidity Index.

**Table 3:** Propensity score-matched analysis of treatment recommendations in patients seen at the Unidisciplinary Spine Clinic (USC) and Multidisciplinary Spine Clinic (MSC).

Parameter	USC (*n* = 437)	MSC (*n* = 437)	*p*-Value
First treatment recommendation			
Injection (%)	44 (10.1)	84 (19.2)	<0.001
Physical therapy (%)	173 (39.6)	189 (43.2)	0.303
Surgery (%)	57 (13.0)	28 (6.4)	0.001
Medically cleared (%)	80 (18.3)	70 (16.0)	0.419
No recommendation (%)	13 (3.0)	16 (3.7)	0.706
Lost to follow-up (%)	70 (16.0)	50 (11.4)	0.062
Time to first treatment order (median days, IQR)			
Injection	28 (12, 64)	19 (0, 45)	<0.001
Physical therapy	0 (0, 21)	0 (0, 21)	0.360
Surgery	4 (0, 28)	5 (0, 56)	0.001
Time to first treatment occurrence (median days, IQR)			
Injection	54 (33, 84)	43 (21, 67)	<0.001
Surgery	43 (25, 85)	43 (26, 65)	0.009

IQR: Interquartile range.

## Data Availability

The data are available.
